# Biodegradability of polyethylene by bacteria and fungi from Dandora dumpsite Nairobi-Kenya

**DOI:** 10.1371/journal.pone.0198446

**Published:** 2018-07-06

**Authors:** Christabel Ndahebwa Muhonja, Huxley Makonde, Gabriel Magoma, Mabel Imbuga

**Affiliations:** 1 Pan African University Institute of Science, Technology and Innovation, Nairobi, Kenya; 2 Department of Pure & Applied Sciences, Technical University of Mombasa, Mombasa, Kenya; 3 Jomo Kenyatta University of Agriculture and Technology, Nairobi, Kenya; Babasaheb Bhimrao Ambedkar University, INDIA

## Abstract

This study aimed at isolating and identifying bacteria and fungi with the capacity to degrade low density polyethylene (LDPE). The level of biodegradation of LDPE sheets with bacterial and fungal inoculums from different sampling points of Dandora dumpsite was evaluated under laboratory conditions. Incubation of the LDPE sheets was done for sixteen weeks at 37°C and 28°C for bacteria and fungi respectively in a shaker incubator. Isolation of effective candidates for biodegradation was done based on the recorded biodegradation outcomes. The extent of biodegradation on the polyethylene sheets was assessed by various techniques including weight loss analysis, Fourier Transform Infrared Spectroscopy (FTIR) and GC-MS. Fourier Transform Infra-Red spectroscopy (FTIR) analysis revealed the appearance of new functional groups attributed to hydrocarbon degradation after incubation with the bacteria and fungi. Analysis of the 16S rDNA and 18S rDNA sequences for bacteria and fungi respectively showed that bacteria belonging to genera *Pseudomonas*, *Bacillus*, *Brevibacillus*, *Cellulosimicrobium*, *Lysinibacillus* and fungi of genus *Aspergillus* were implicated as polyethylene degraders. An overall analysis confirmed that fungi are generally better degraders of polyethylene than bacteria. The highest fungal degradation activity was a mean weight reduction of 36.4±5.53% attributed to *Aspergillus oryzae* strain A5, 1 (MG779508). The highest degradation activity for bacteria was a mean of 35.72± 4.01% and 20.28± 2.30% attributed to *Bacillus cereus* strain A5,a (MG645264) and *Brevibacillus borstelensis* strain B2,2 (MG645267) respectively. Genus *Aspergillus*, *Bacillus* and *Brevibacillus* were confirmed to be good candidates for Low Density Poly Ethene bio-degradation. This was further confirmed by the appearance of the aldehyde, ether and carboxyl functional groups after FTIR analysis of the polythene sheets and the appearance of a ketone which is also an intermediary product in the culture media. To improve this degrading capacity through assessment of optimum conditions for microbial activity and enzyme production will enable these findings to be applied commercially and on a larger scale.

## Introduction

Approximately 140 million tons of plastics are produced every year and high amounts find themselves in the ecosystem as industrial waste products [1]. About 30% of the plastics are used worldwide for packaging of foods, pharmaceuticals, cosmetics, detergents and chemicals and this is still expanding at a high rate of 12% p.a [2]. Plastics have replaced paper and other cellulose-based products for packaging because they have better tensile strength, lightness, resistance to water and microbial attack. Commonly used plastics have been categorized as polyethylene (LDPE, MDPE, HDPE and LLDPE), polypropylene (PP), polystyrene (PS) and polyvinyl chloride (PVC) [3]. Low Density Polyethylene belongs to thermoplastics class [4] and is believed to have non-degradable nature due to its hydrophobic backbone [5]. This has forced many governments to come up with measures to curb this menace. Bangladesh, for instance, imposed a ban on plastic bags in March 2002 following flooding caused by blockage of drains. The management of solid waste in Kenya has been poor and even worst in major cities such as Nairobi [6]. The consumption of plastics in the country has increased to 4,000 tons per annum of polyethylene bags which together with hard plastics end up scattered in the environment creating “the plastics menace”. Kenya through the National Environmental Management Authority (NEMA) has embraced the 3Rs: Reduce, Re-use and Recycle concept of solid waste management but this has not addressed the problem of polyethylenes which remain scattered in the environment as recorded by [7]. Most recently, the Kenyan ministry of Natural Resources, through the NEMA imposed a ban on the use of polyethylene carrier bags from 28^th^ August 2017 in an attempt to reduce the amount of polyethylene being released into the environment [8]. Despite this move, polyethylene continues to be used especially in packaging of goods.

Biodegradation can be defined as the decomposition of substances through microbial activity. This is a complex process which involves several steps [9]: bio-deterioration (the combined action of microbial communities and abiotic factors to fragment the materials into tiny fractions), depolymerization (Microorganisms secrete enzymes and free radicals able to cleave polymer into oligomers, dimers and monomers, assimilation (some molecules are recognized by receptors of microbial cells and can go across the plasma membrane) and mineralization (simple molecules as CO_2_, N_2_, CH_4_, H_2_O and different salts from intracellular metabolites that are completely oxidized are released) [10]. Bacteria and fungi have been implicated in this process albeit slow rates. According to [11], *Pseudomonas species* are most highly implicated in the biodegradation of LDPEs. They isolated *Pseudomonas citronellolis* EMBS027 which had 17.8% weight reduction on polyethylene sheets. [12] isolated *Brevibaccillus borstelensis* strain 707 which upon 30 days incubation at 50°C reduced the gravimetric and molecular weights of polyethylene sheets by 11 and 30% respectively. Fungal isolates: *Fusarium sp*. AF4, *Aspergillus terreus* AF5 and *Penicillum sp*. AF6 were found attached to Polyethylene sheets mixed with sewage sludge for ten months [9]. Ability of *Bacillus subtilis* to degrade polyethylene was also demonstrated by [13] in the presence and absence of bio-surfactants. [14] isolated several fungal genera that were able to degrade polyethylene sheets with *Aspergillus niger* showing the highest weight reduction of 4.32%.

Fourier Transform Infrared spectroscopy (FT-IR) is used to indicate the map of the identified compounds on the surface of the sample and document via collection of spectra [4]. Spectra of sheets obtained from four different LDPE samples by [15] showed introduction of some new peaks after the period of biodegradation with peaks of carbonyl groups (1720 cm-1), CH3 deformation (1463 cm-1) and C = C conjugation band (862 cm-1). The weight loss, percentage of elongation and change in tensile strength can be applied to measure the physical changes of the polyethylene. The products from polyethylene degradation are also characterized using techniques such as Gas Chromatography-Mass Spectrometry (GC-MS) [4, 16].

This study aimed at isolating and identifying bacteria and fungi that have the capacity to degrade low-density polyethylene. Evidence of biodegradation was based on weight loss, FT-IR and GC-MS outcomes which confirmed that microbes are capable of degrading LDPE.

## Materials and methods

### Study site

Dandora dumpsite is about 8 km away from Nairobi city and is adjacent to the heavily populated low income estates of Dandora, Korogocho, Baba Dogo and Huruma. It is home to a 1\4 a million people and it sits on over 30 solid acres. It contains mixed wastes that include domestic wastes from homes, expired goods, agricultural wastes, and hospital wastes most of which are or come in polyethylene carriers. Polythene bags made up 225 tons of the 2000 tons of waste in Nairobi in a single day by the year 2006. The sampling points were as follows: **A:** S01°14.633 E036 °54.063 Elevation 1578m, 21.48Km SW, **B:** S01°14.652 E036 °54.031 Elevation 1589m, 21.55Km SW, **C****:** S01°14.661 E036 °53.977 Elevation 1590m 21.63km SW **D:** S01°14.688 E036 °53.986 Elevation 1594m 21.65km SW and **E:** S01°14.710 E036 °53.977 Elevation 1596m 21.69km SW

### Sample collection and preparation of medium for LDPE degrading bacteria

A randomized block design was used to identify points for sample collection. Soil samples were collected randomly from the five selected sampling blocks of the dumpsite. The samples from each sampling block were collected at 5 different points of 1m diameter. This resulted in 25 soil samples collected. Soil was aseptically scooped from and adjacent to buried polyethylene materials at 5 cm depth below the litter layer. On-the-site temperature was recorded in order to ascertain the in-situ biodegradation conditions. Samples were kept in Ziploc bags and transported to the Institute of Biotechnology Research (IBR) lab at JKUAT in a cool box. Once in the lab, the pH of the samples was also measured and recorded.

### Preparation of artificial media and incubation

1 g of soil sample was added to 50ml of 0.85% autoclaved normal saline solution to prepare the inoculums. The inoculum was kept at 37°C for 2–3 hr in a shaker incubator before inoculation. Artificial media was prepared i.e. 0.1% (NH_4_)_2_ SO_4_, 0.1% NaNO_3_, 0.1% K_2_HPO_4_, 0.1% KCl, 0.02% MgSO_4_ and 0.001% yeast extract in 1000ml distilled water [17]. LDPE powder weighing 2g was added as the carbon source to each 100 ml of growth medium. 1% of the prepared inoculums was transferred to 200 ml of synthetic medium to prepare growth culture for the LDPE degrading microorganisms. Sheets of Polyethylene (app 3 cm x 3 cm each), weighed, disinfected in 70% ethanol and air-dried for 15 min in an oven were introduced into the synthetic media. Culture flasks for fungal incubation were augmented with 250mg/ml ampicillin to inhibit bacterial growth. All treatments were done in triplicates. Synthetic media with LDPE without inoculum was used as the negative control. All the treatments were incubated in an incubator shaker at 150 rpm for up to 16 weeks.

### Determination of LDPE degrading potential of the bacterial isolates

The LDPE sheets were recovered after different incubation intervals (8 weeks, 12weeks and 16 weeks). Washing of the sheets was done using 2% SDS to remove the bacterial biomass then dried overnight before being weighed. At the end of the incubation period, the structural changes in the LDPE surface was investigated using the EQUINOX 55 FT-IR spectrometer [4, 18, 19].

For each LDPE sheet, a spectrum was taken from 400 to 4000 wave numbers at a resolution of 2 cm-^1^ and over 32 scans. The control was not subjected to any incubation process. The incubation media was subjected to GC-MS analysis. The results were recorded and analyzed.

### Isolation of LDPE degrading microorganisms

Based on the results above, bacterial isolation was done from the culture flasks at the end of the incubation period. Isolation was only carried out from those incubation flasks that had shown indications of biodegradation based on weight changes, FTIR and GC-MS outcomes.

For bacterial isolation, a loopful of culture from the synthetic media was put on a nutrient agar plate, a spreader was used to spread it till dry. Incubation was done overnight at 37°C or up to three days for slow growing bacteria. The mixed cultures of bacteria were continually sub-cultured to obtain pure bacterial cultures. They were stored at– 20°C and at -80°C in 15% glycerol slants.

For fungal isolation, a drop of the inoculum was put and spread till dry on Potato Dextrose Agar (PDA) plates and incubated for five days. The various fungi were isolated based on morphology and sub-cultured continually to obtain pure fungal cultures.

### Identification of isolates

Total genomic DNA for 16S rDNA amplification was isolated from bacterial cells grown overnight by means of a standard protocol [20]. Amplification of the 5' end of the 16S rDNA gene was performed with universal primers forward primer (8-F) 5'-AGAGTTTGATYMTGGCTCAG- 3' and reverse primer (1942R) 5'- GGTTACCTTGTTACGACTT-3' [21]. For fungal isolates, CTAB method of genomic DNA extraction was used [22]. Primer pair 566-F:5' - CAGCAGCCGCGGTAATTCC - 3' and for 1200-R:5'- CCCGTGTTG AGTCAAATTAAGC- 3' which amplify on average a 650 bp long fragment from the V4 and V5 regions [23] were used. The similarity of the sequences obtained against known deposited 16S rDNA and 18S rRNA sequences from closely related bacteria and fungi respectively was tested with BLASTN 2.2.1 upon sequence editing with Chromas Pro version 2.6.2.

## Results

### Weight loss of the polyethylene sheets

**Fig 1:**
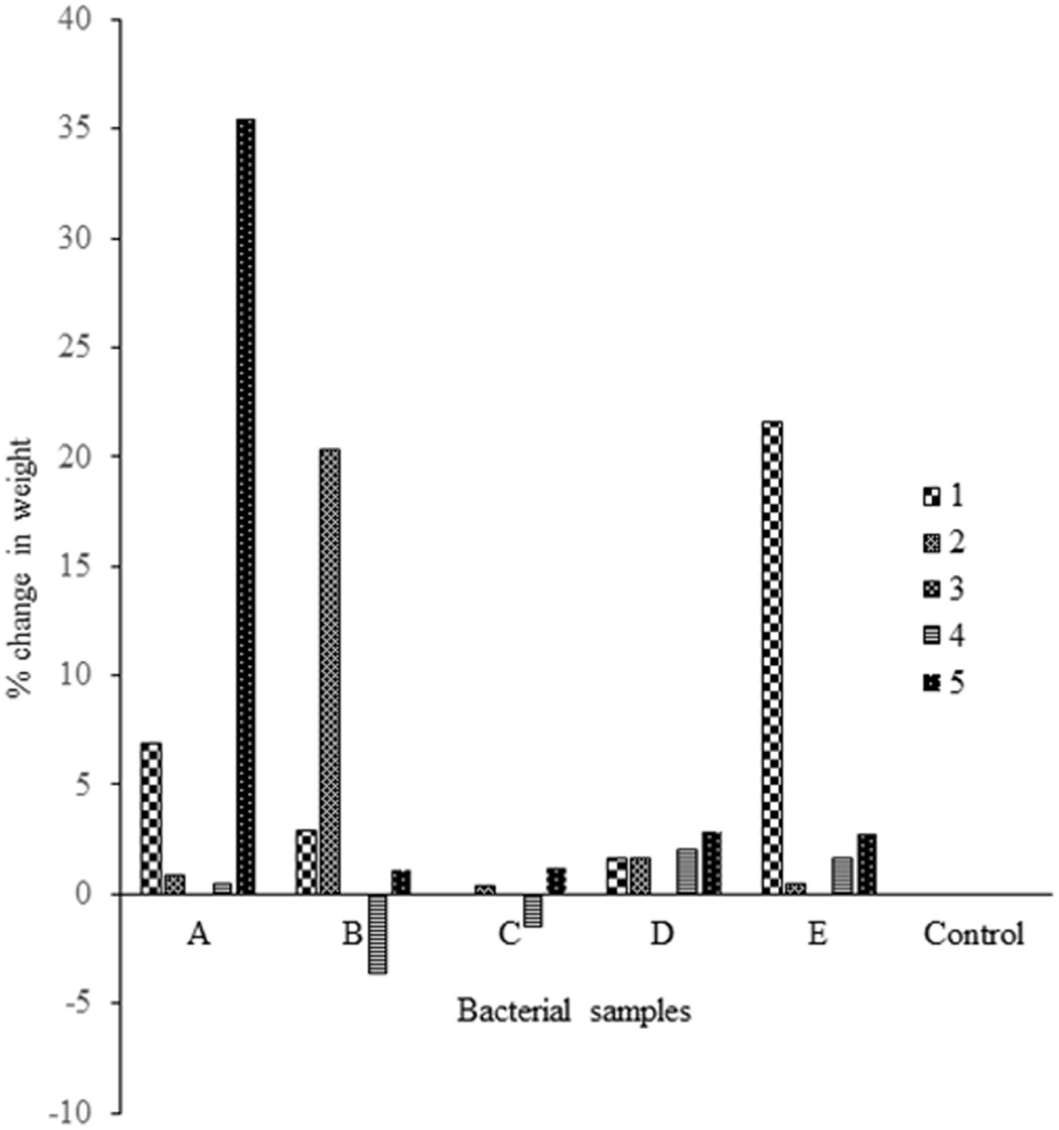
% mean weight reduction of the 30 micron polyethylene sheet incubated with 25 bacterial sub-samples which were found to contain specific bacterial isolates after incubation at 37 °C for sixteen weeks. Each mean weight represents the average of three replicates ± SE.

**Fig 2:**
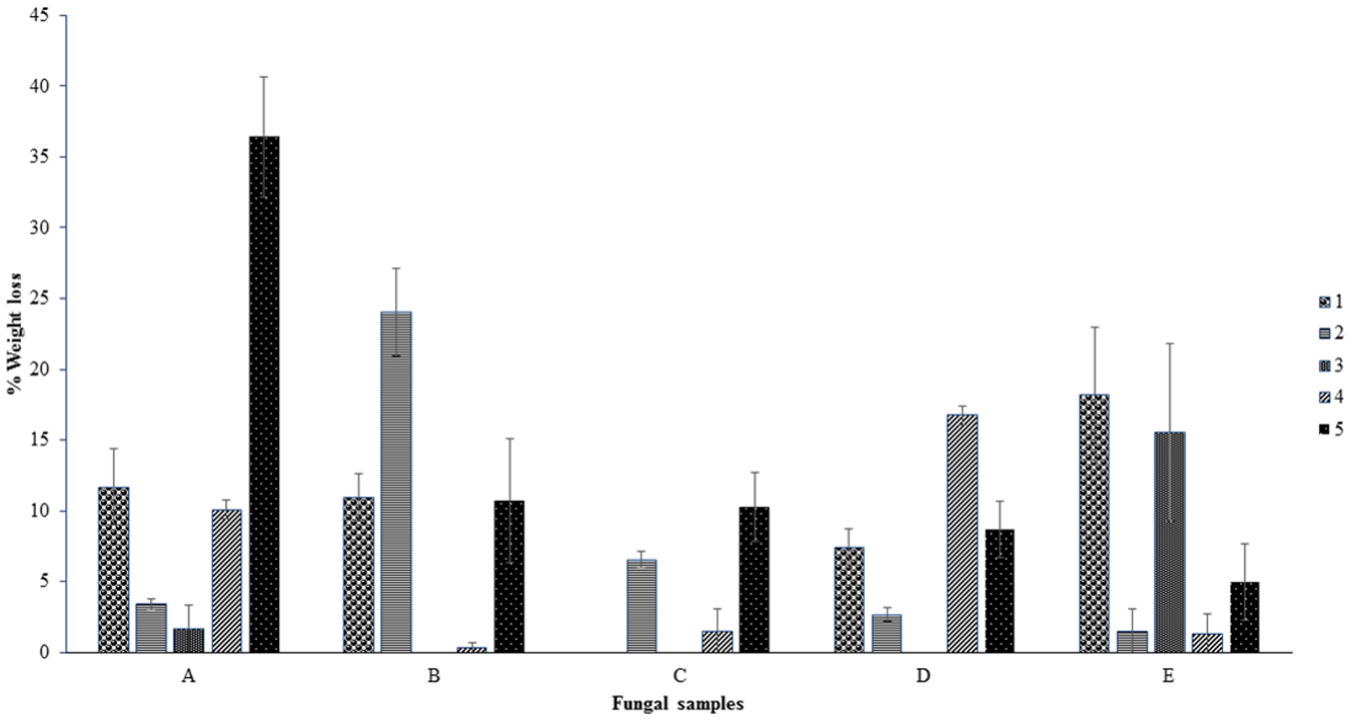
% mean weight reduction of the 30 micron polyethylene sheets incubated with 25 fungal sub-samples which were found to contain specific isolates after incubation at 28 °C for sixteen weeks. Each mean weight represents the average of three replicates ± SE.

**Fig 3:**
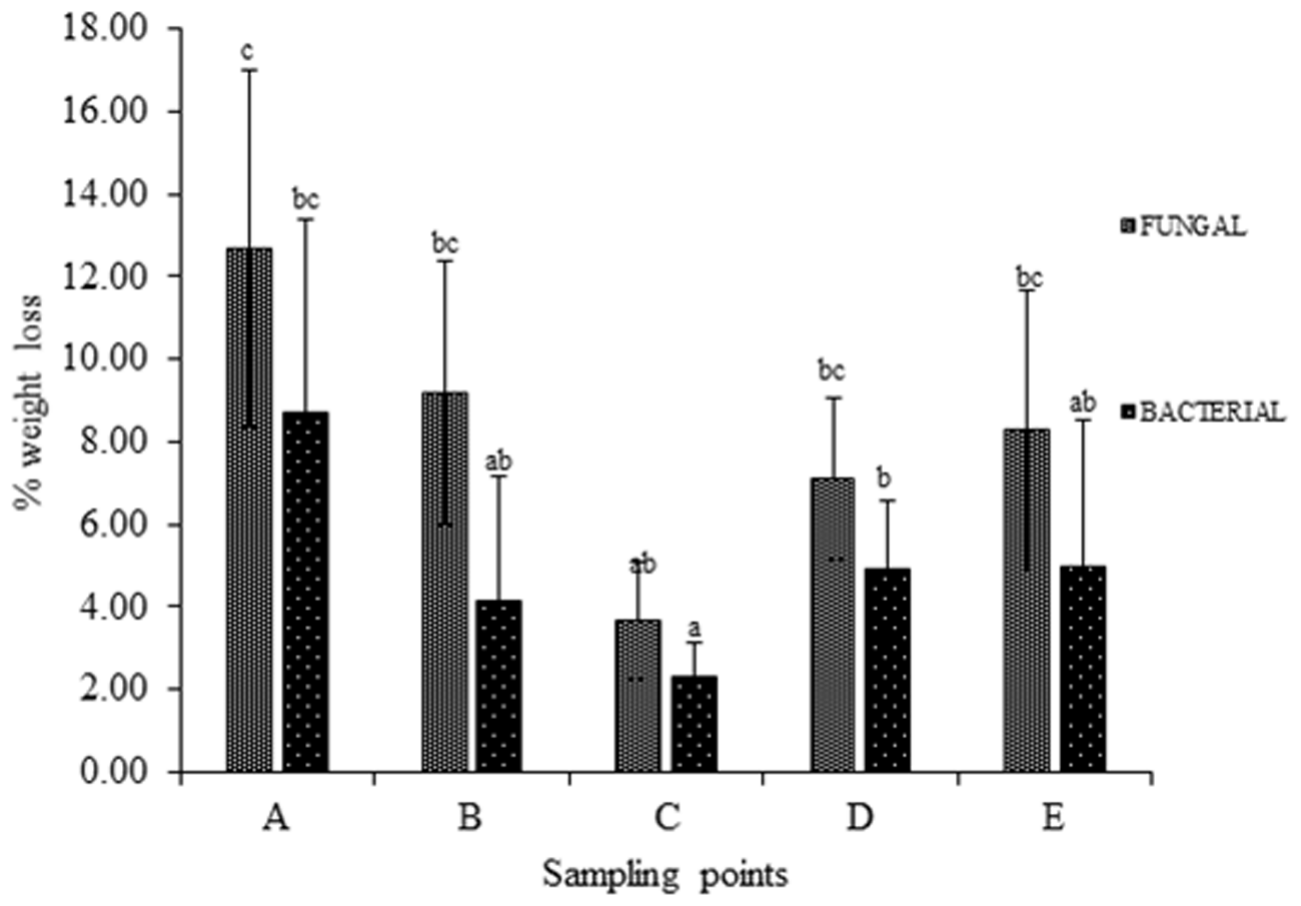
% reduction in mean weight of the 30 micron polyethylene sheets after sixteen weeks incubation with fungal and bacterial inoculums at 28 °C and 37°C respectively. Each mean weight represents the average of five sample means± SE. Means with same letters are not significantly different using Fisher’s Protected Least Significant test at (P<0.05).

**Fig 4:**
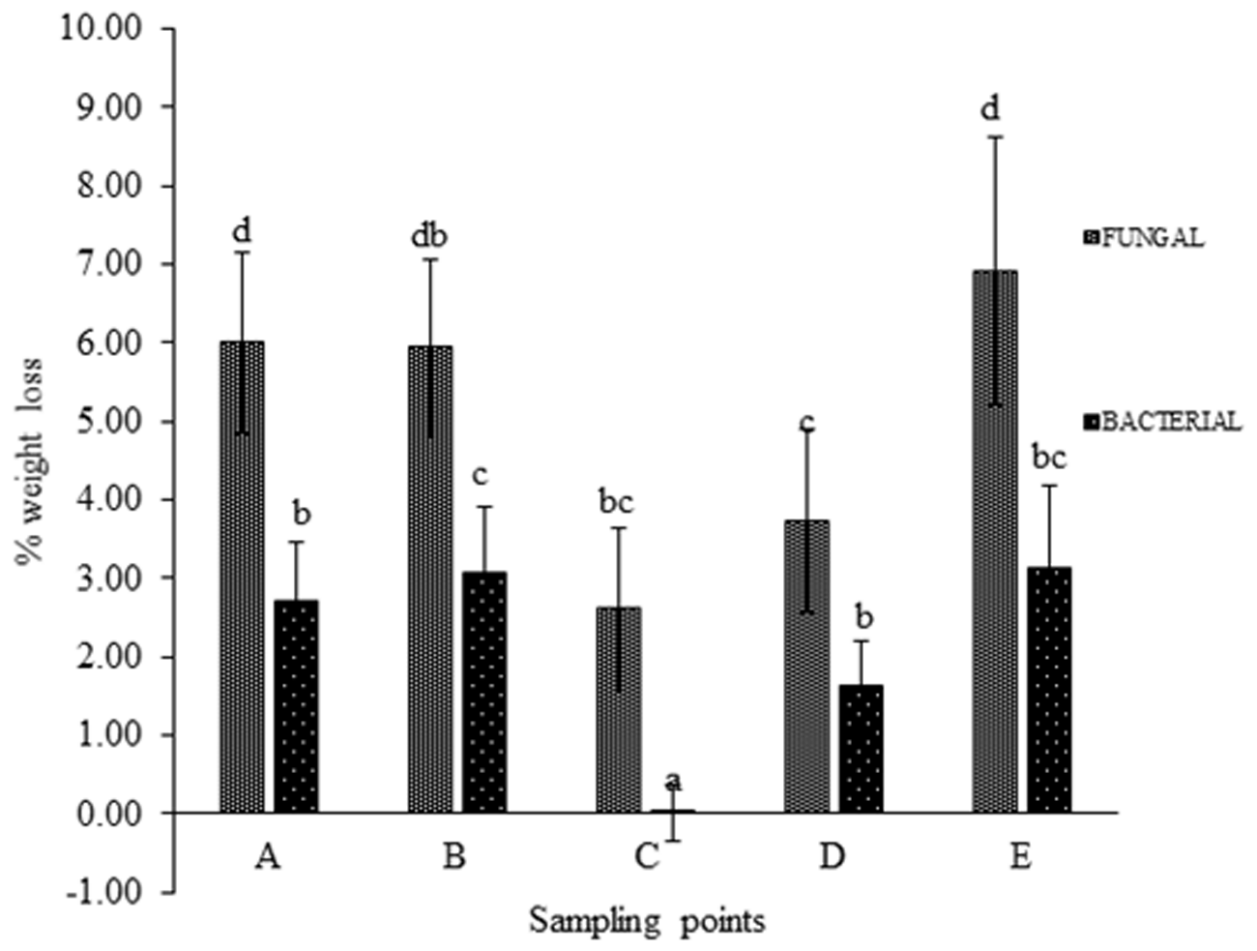
% reduction in mean weight of 40 micron polyethylene sheets after sixteen weeks incubation with fungal and bacterial inoculums at 28 °C and 37°Crespectively. Each mean weight represents the average of five sample means ± SE. Means with same letters are not significantly different using Fisher’s Protected Least Significant test at (P<0.05).

**Fig 5:**
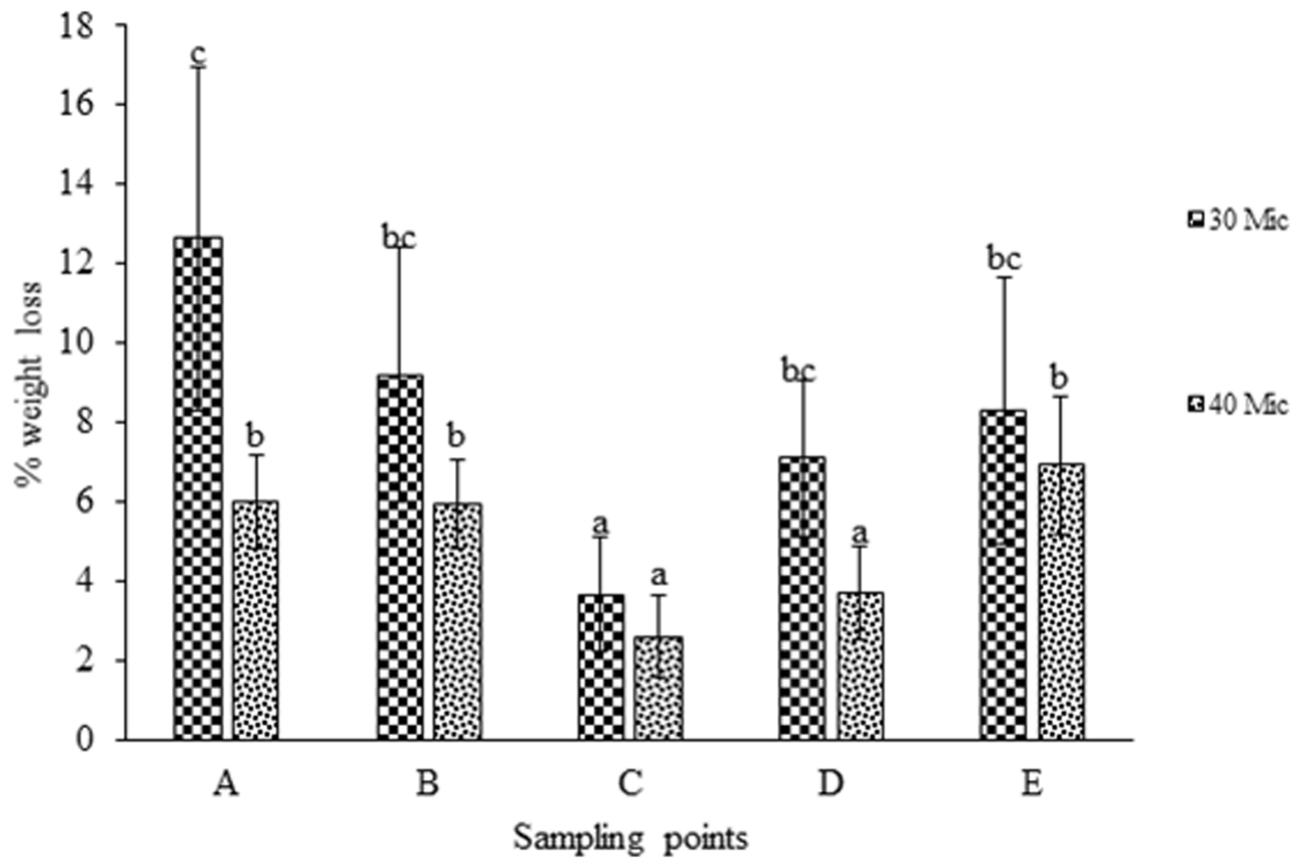
% mean weight reduction of the 30 micron and 40 micron polyethylene sheets incubated with fungal inoculums from sampling points A, B, C, D and E at 28 °C for sixteen weeks. Each mean weight represents the average of five sample means ± SE. Means with same letters are not significantly different using Fisher’s Protected Least Significant test at (P<0.05).

**Fig 6:**
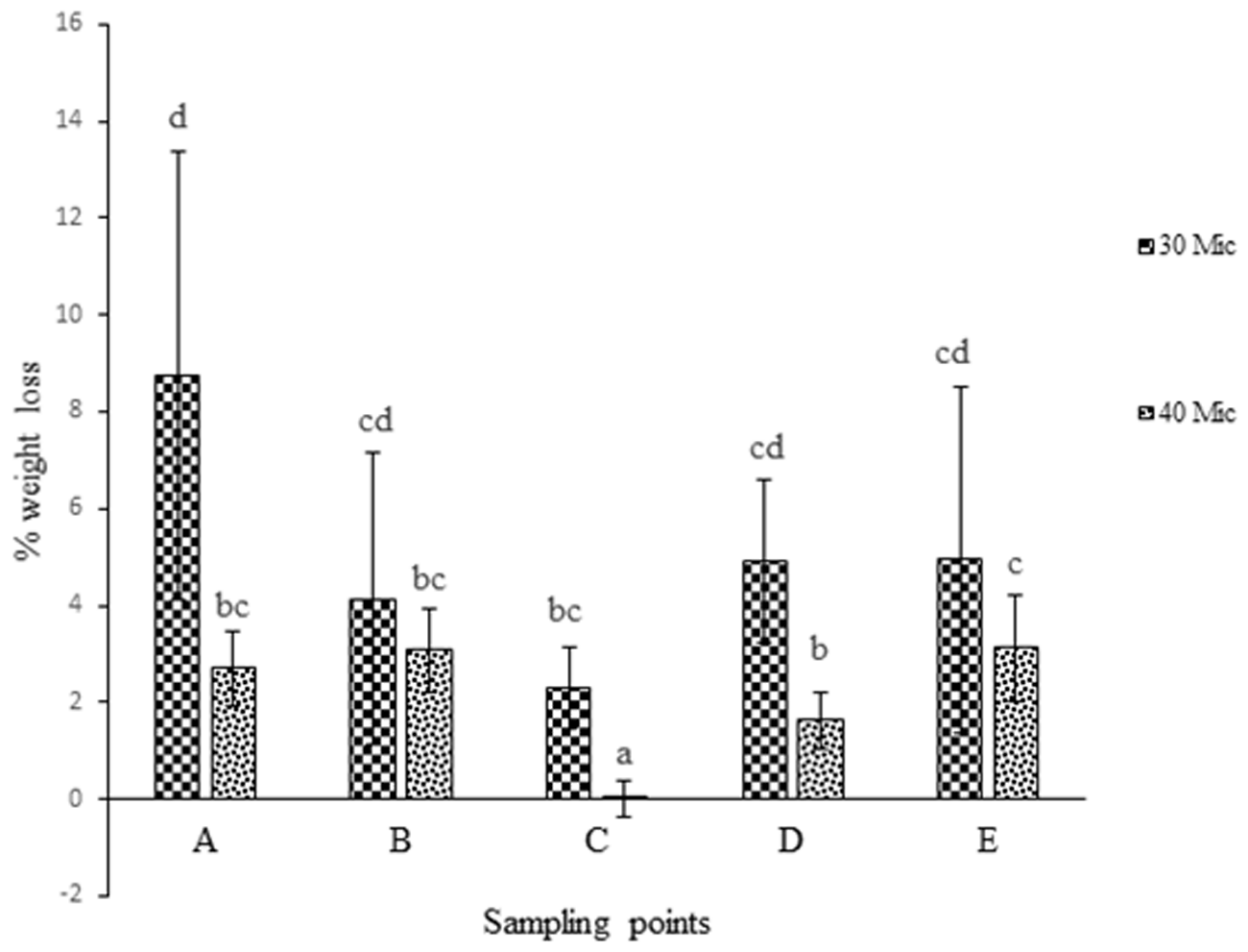
A comparison between % mean weight reduction of the 30 micron and 40 micron polyethylene sheets incubated with bacterial inoculums from sampling points A,B, C,D and E at 37 °C for sixteen weeks. Each mean weight represents the average of five sample means ± SE. Means with same letters are not significantly different using Fisher’s Protected Least Significant test at (P<0.05).

Mean weight changes were computed and used to compare the effectiveness biodegradation between fungi and bacteria as well as between the two sets of polyethylene sheets (30 and 40 microns) (Tables 1 and 2). To get the % change in weight, the formula:

Changeinweight/originalweight×100%=Percentagechangeinweight

was used. Weight loss % analysis was done and represented graphically for bacterial activity on 30 micron polythene (Fig 1), fungal activity on 30 micron polythene (Fig 2), comparative fungal and bacterial activity on 30 micron polythene (Fig 3), comparative fungal and bacterial activity on 40 micron polythene (Fig 4), comparative fungal activity on the 30 and 40 micron polythene (Fig 5) and a comparative bacterial activity on the 30 and 40 micron polythene (Fig 6).

**Table 1:** 30 micron polythene % reduction in weight (mg) following sixteen weeks incubation with fungal and bacterial isolates at 28°C and 37 °C respectively. A negative mean indicates there was a weight gain instead of loss.

No.	Fungal Sample	Mean wt loss %	S.E	Bacterial Sample	Mean wt. loss %	S.E
	A1	11.44^e^	1.58	A1	6.85^cd^	0.67
	A2	3.71^abc^	0.39	A2	0.83^b^	0.18
	A3	3.38^abc^	0.56	A3	0.37^b^	0.37
	A4	10.08^de^	0.71	A4	0.45^b^	0.79
	A5	36.40^g^	5.53	A5	35.72^g^	4.01
	B1	10.79^e^	0.99	B1	2.88^bc^	0.38
	B2	24.10^f^	3.26	B2	20.28^e^	2.30
	B3	0.19^a^	0.19	B3	0.37^b^	0.06
	B4	0.31^a^	0.19	B4	-4.88^a^	1.23
	B5	14.00^e^	1.79	B5	1.82^bc^	0.30
	C1	6.55^bcd^	0.07	C1	0.05^b^	0.05
	C2	2.28^ab^	0.24	C2	1.20^bc^	0.24
	C3	3.37^abc^	0.28	C3	0.13^b^	0.13
	C4	0.33^a^	0.20	C4	2.33^bc^	0.38
	C5	10.27^e^	0.98	C5	1.11^bc^	0.10
	control	0^a^	0	control	0^b^	0
	D1	2.65^ab^	0.94	D1	2.48^bc^	0.40
	D2	2.08^ab^	0.59	D2	2.62^bc^	0.34
	D3	0^a^	0	D3	0^b^	0
	D4	16.77^e^	0.57	D4	1.74^bc^	0.27
	D5	8.62^e^	0.21	D5	4.66^cd^	0.32
	E1	18.43^f^	2.20	E1	20.05^e^	4.21
	E2	1.564^ab^	0.17	E2	0^b^	0
	E3	18.15^f^	2.20	E3	1.01^b^	0.10
	E4	1.55^ab^	0.80	E4	0.09^b^	0.10
	E5	5.65^bcd^	1.024	E5	3.57^bcd^	0.31

Means with same superscript letters in the same column are not significantly different while means with different superscript letters are significantly different using Fisher’s Protected Least Significant test at (P<0.05). The superscript letters are arranged in ascending order with ‘a’ indicating the least significant mean change in weight while ‘g’ indicates the most significant mean change in weight. Means that have a shared range have a shared superscript letter.

**Table 2:** 40 micron polythene % reduction in weight (mg) upon sixteen weeks incubation with various fungal and bacterial isolates at 28°C and 37 °C respectively.

No.	Fungal Isolate	Mean wt. loss %	SE	Bacterial Isolate	Mean wt. loss %	SE
1	A1	7.73^d^	0.18	A1	1.15^ab^	0.23
2	A2	4.08^bc^	0.12	A2	2.76^bcd^	0.36
3	A3	4.25^bc^	0.14	A3	1.93^abc^	0.33
4	A4	7.43^d^	0.59	A4	3.65^cd^	0.41
5	A5	11.00^ef^	0.97	A5	6.43^ef^	0.57
6	B1	10.09^e^	0.25	B1	10.18^gh^	0.51
7	B2	5.03^c^	0.15	B2	8.46^fg^	1.22
8	B3	0^a^	0	B3	0^a^	0
9	B4	7.86^d^	0.18	B4	2.98^bcd^	0.30
10	B5	7.03^d^	0.53	B5	2.94^bcd^	0.50
11	C1	0^a^	0	C1	1.12a^b^	0.62
12	C2	6.67^d^	0.34	C2	8.27^fg^	1.13
13	C3	0a	0	C3	0^a^	0
14	C4	3.44^b^	0.29	C4	2.77^bcd^	0.19
15	C5	12.34^f^	0.27	C5	4.35^de^	0.24
16	control	0^a^	0	control	0^a^	0
17	D1	7.87^d^	0.94	D1	4.44^de^	0.55
18	D2	2.94^b^	0.41	D2	3.28^bcd^	0.62
19	D3	0^a^	0	D3	0^a^	0
20	D4	17.01^h^	0.45	D4	11.13^h^	3.26
21	D5	10.41^e^	0.38	D5	7.24^f^	0.97
22	E1	7.99^d^	0.37	E1	1.55^abc^	0.21
23	E2	4.20^bc^	0.16	E2	0^a^	0
24	E3	0^a^	0	E3	2.09^abcd^	0.20
25	E4	10.80^e^	0.36	E4	8.13^fe^	0.30
26	E5	14.36^g^	1.54	E5	6.33^ef^	0.33

Means with same superscript letters in the same column are not significantly different while means with different superscript letters are significantly different using Fisher’s Protected Least Significant test at (P<0.05). The superscript letters are arranged in ascending order with ‘a’ indicating the least significant mean change in weight while ‘h’ indicates the most significant mean change in weight. Means that have a shared range have a shared superscript letter.

### FT-IR (Fourier Transform Infra-Red) outcomes

**Fig 7:**
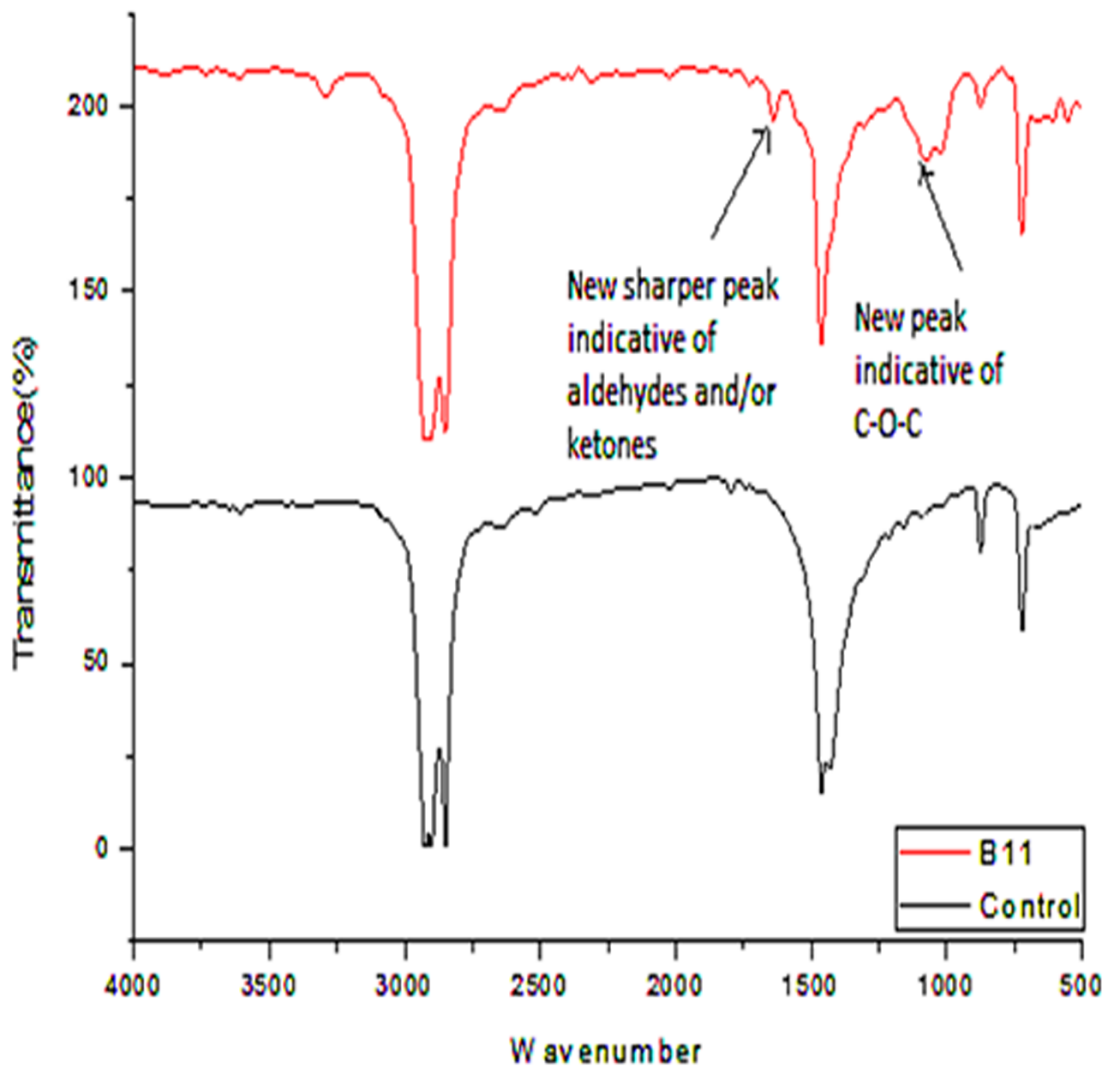
FT-IR spectra of polyethylene sheet from sample B1, 1 incubated with bacterial inoculum of *Pseudomonas putida* strain B1, 1a (MG645383) and the control incubated at 37 °C for sixteen weeks.

**Fig 8:**
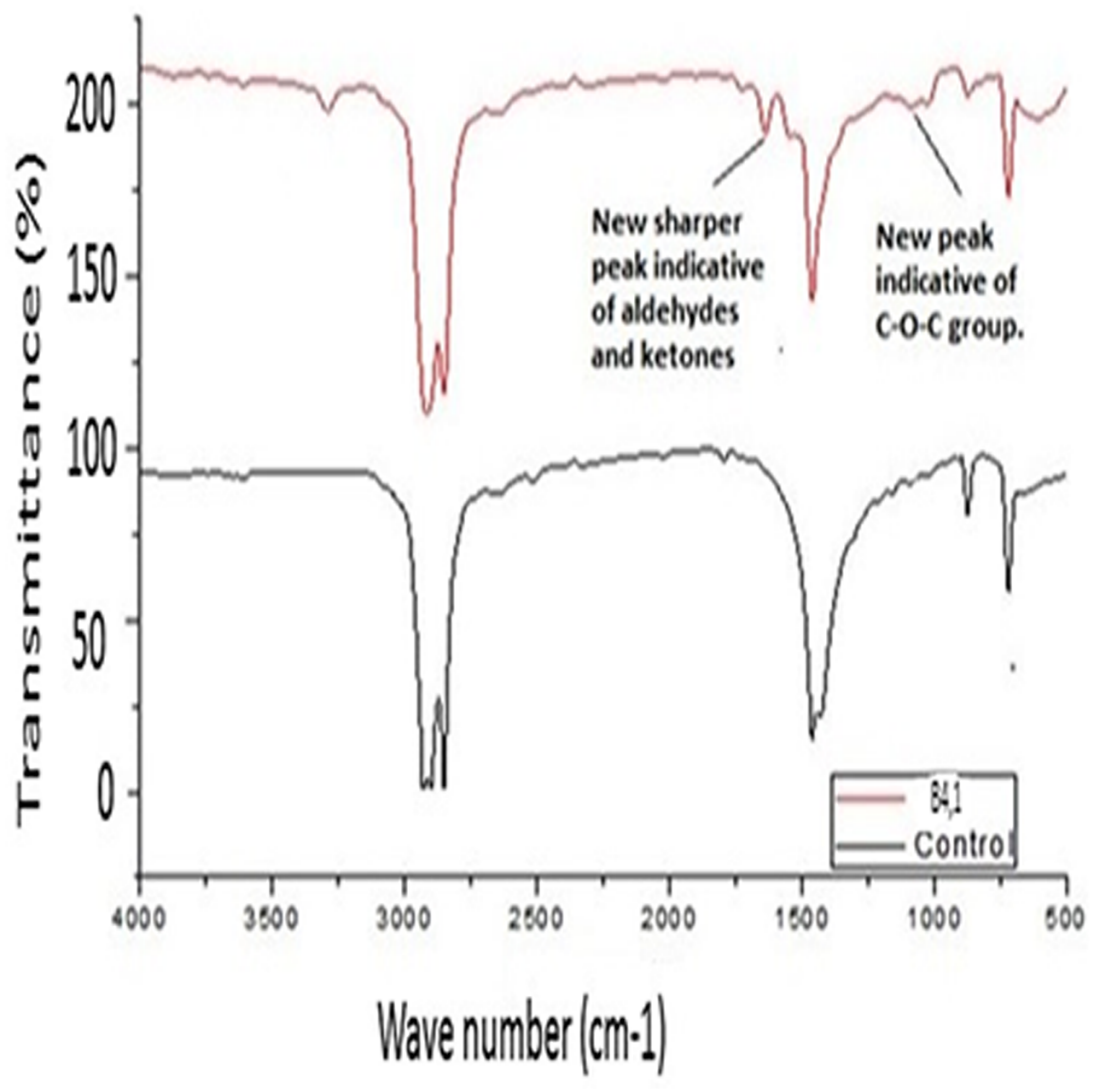
FT-IR spectra of polyethylene sheet from sample B,4,1 with bacterial inoculum of *Bacillus cereus* strain A1, a (MG645253) and the control incubated at 37 °C for sixteen weeks.

**Fig 9:**
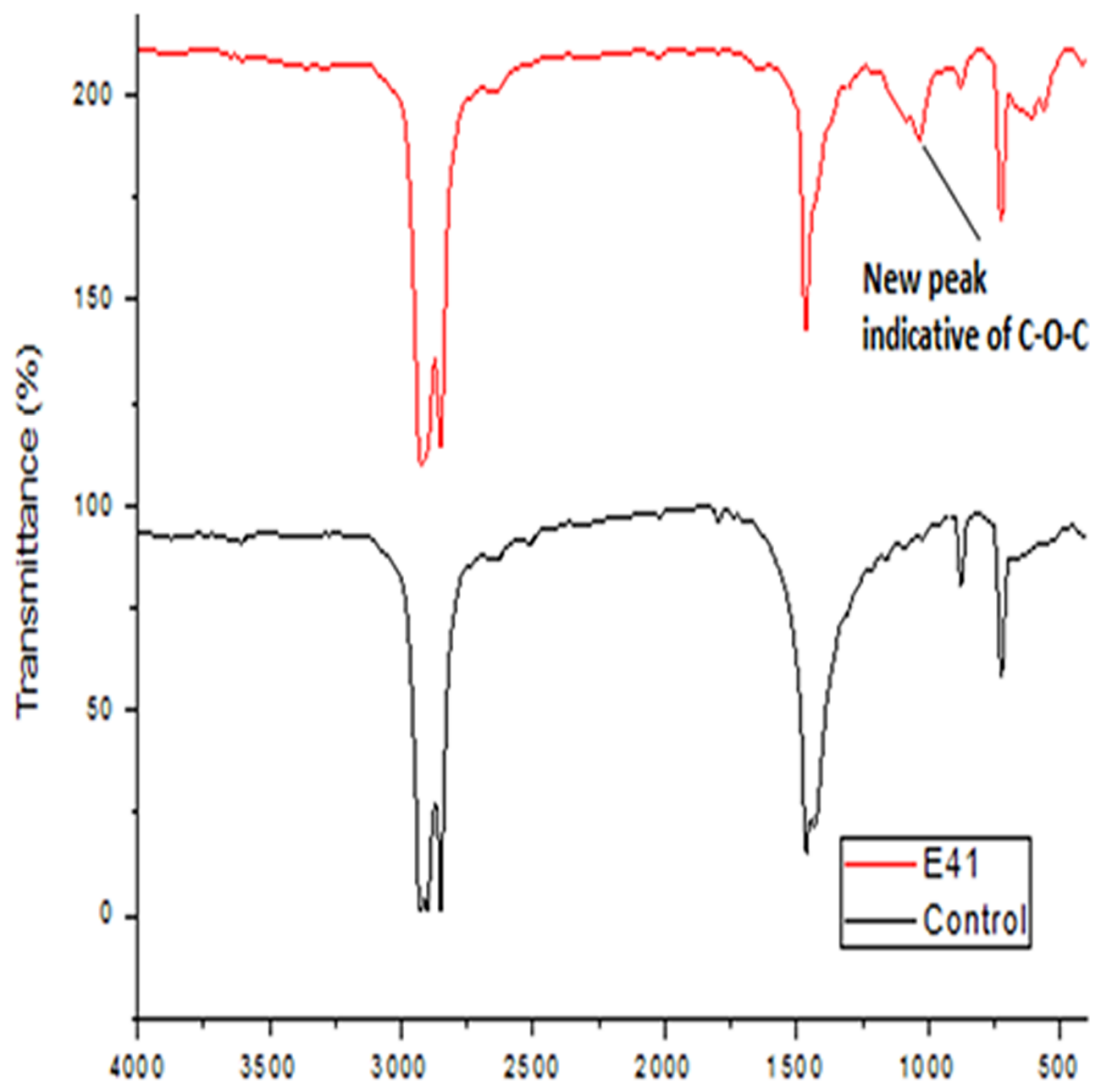
FT-IR spectra of polyethylene sheet from sample E4, 1 with fungal inoculum of *Aspergillus nidulans* strain E4,1(MG779504) and the control incubated at 28 °C for sixteen weeks.

**Fig 10:**
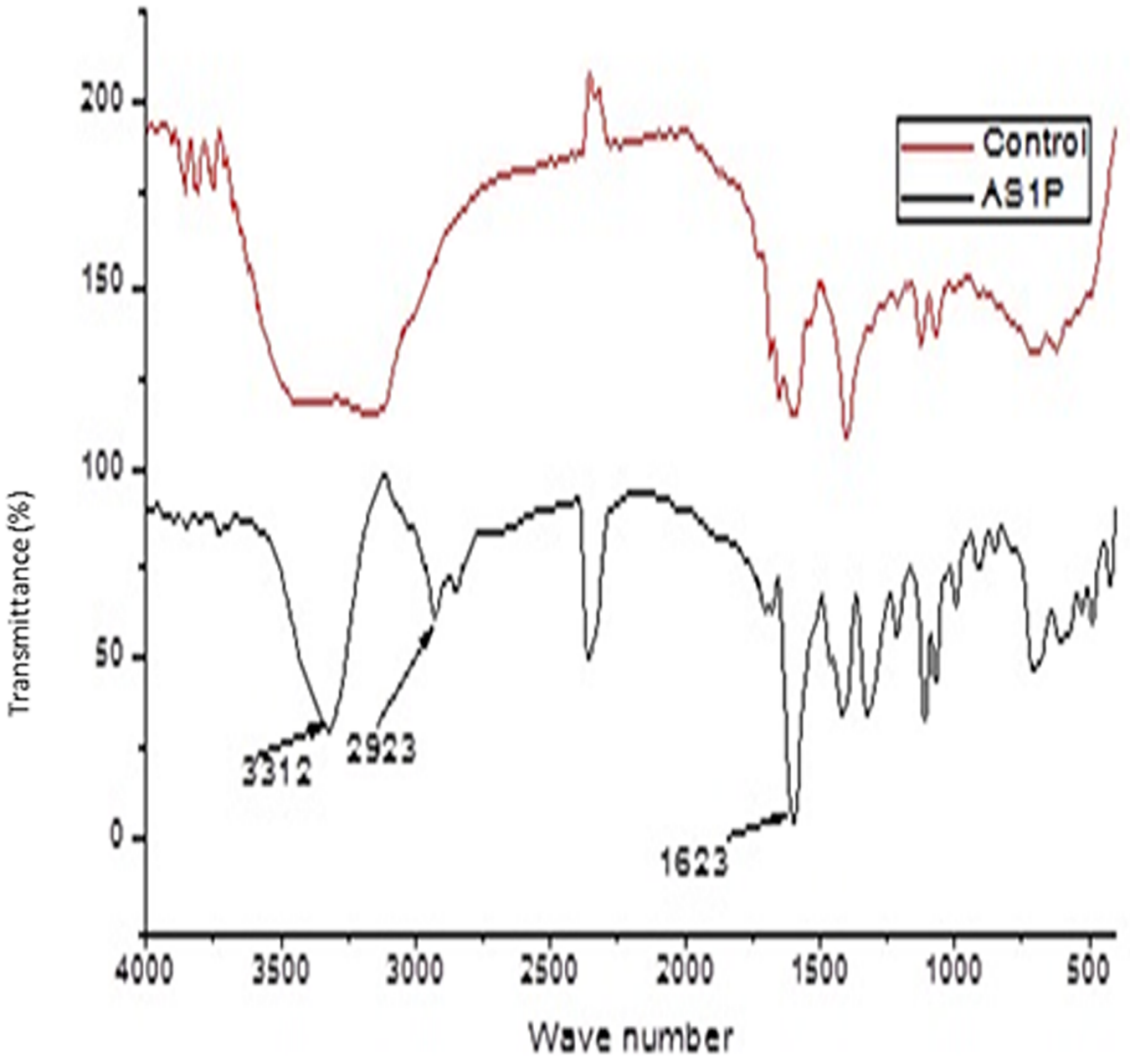
FT-IR spectra of polyethylene powder from sample A5, 1 with fungal inoculum of *Aspergillus oryzae* strain A5,1 (MG779508) and the control incubated at 28 °C for sixteen weeks.

**Fig 11:**
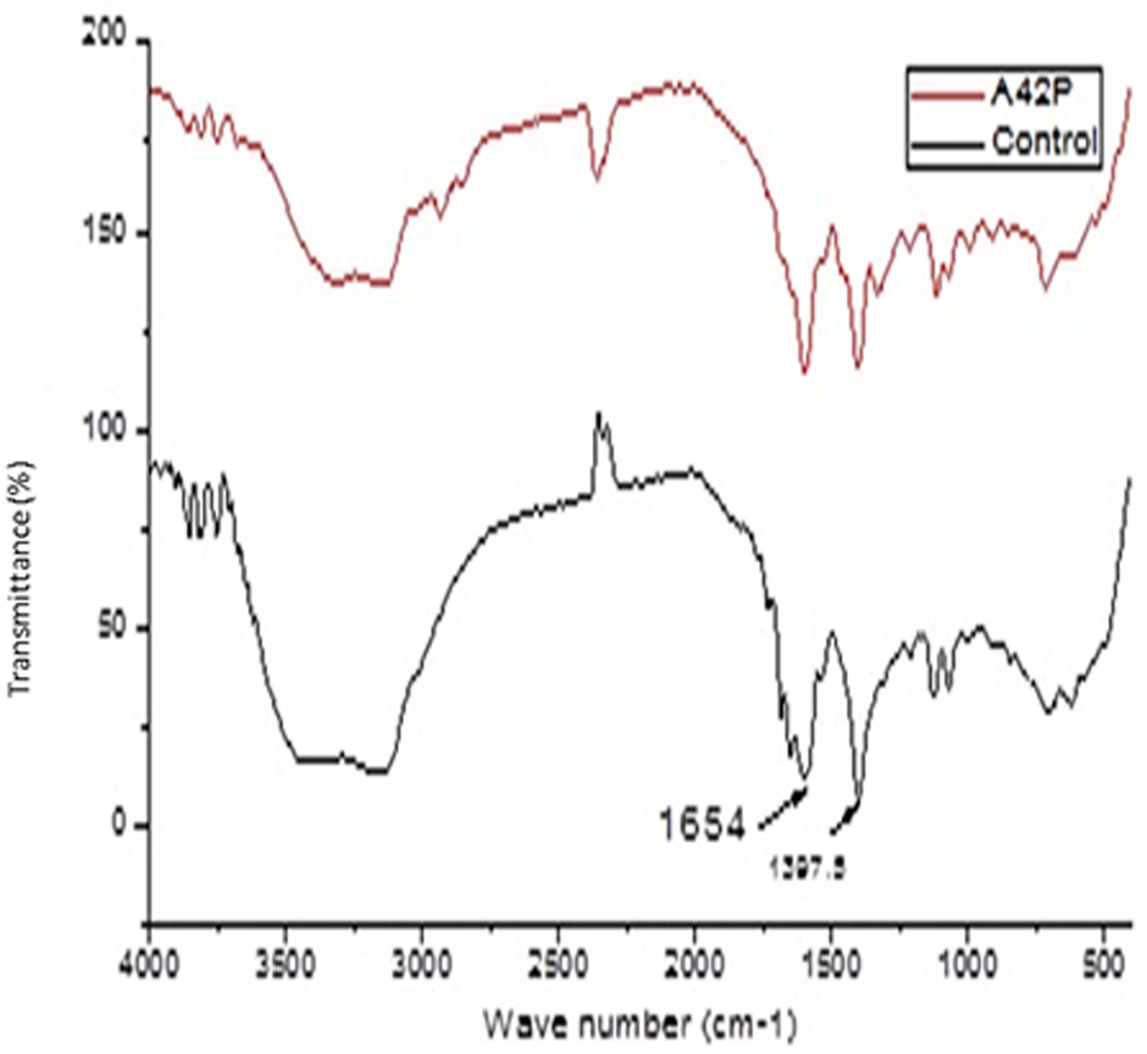
FT-IR spectra of polyethylene powder from sample A4, 2 with fungal inoculum of *Aspergillus flavus* strain A4,2 (MG779506) and the control incubated at 28 °C for sixteen weeks.

Spectral figures indicating formation of new functional groups as a result of new peaks on the polythene sheets and the control (incubated without inoculum) sheets were superimposed as follows (Figs 7–11).

### GC-MS results

The gas chromatogram output from the sample of *Aspergillus oryzae* (MG779508) indicated the compounds and their retention times (Table 3).

**Table 3:** Compounds identified after the culture media from the fungal incubation of sample A5, 2 for 16 weeks was subjected to GC-MS.

Compound ID	Retention time (minutes)
4,4-Dimethyl-2-pentene	4.209
4,6-Octadiyn-3-one, 2-methyl	4.750

### Morphological characterization

**Fig 12:**
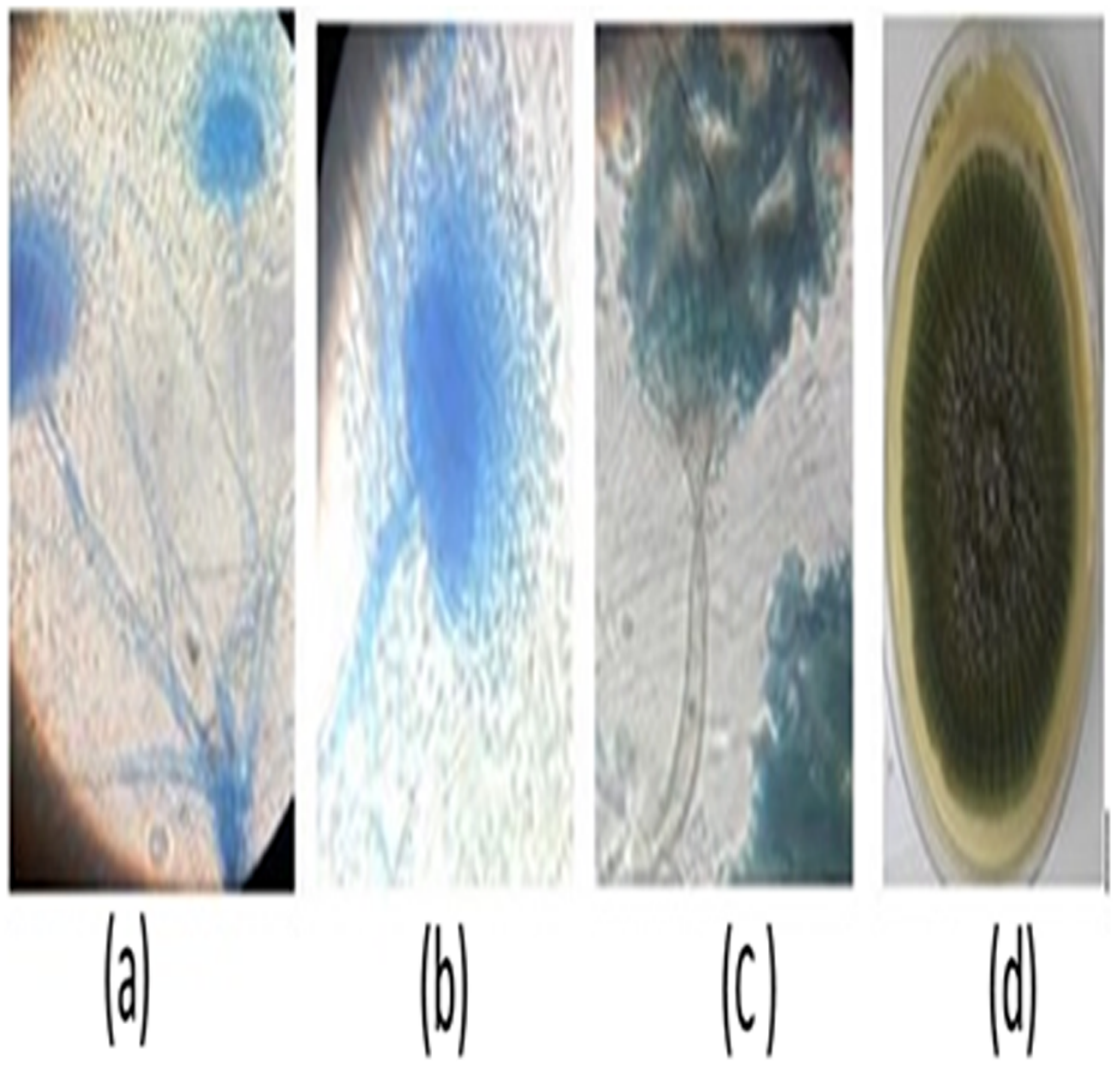
Microscopic examination outcomes from lactophenol blue staining of *Aspergillus fumigatus* strain B2, 2 (MG779513) with a and b indicating erect conidia and aerial conidiophores. The conidiophores are branched with bottle-shaped phialides at the tip of the conidiophores producing spores while d is a photo of 5-day old fungal growth. c has a simple conidiophore terminated by flask shaped phialides where spores are produced in chains at the tip end characteristic of genus *Penicillium*.

Based on the above indicators of Polyethylene biodegradation as shown by the structural changes on in FTIR spectra (Figs 7–11), the weight reduction of the LDPE sheets indicated by the mean weight changes in graphical Figs 1–6 and the formation of intermediate degradation products in Table 3, the respective incubation flasks containing synthetic media plus inoculum from soil were subjected to isolation of bacteria and fungi. A total of 30 bacterial isolates were isolated. Among this 7 were Gram negative while 23 were Gram positive. A total of 26 fungal isolates were isolated. Among these 20 were macroscopically and microscopically profiled to belong to the genus *Aspergillu*s while six were profiled as belonging to the genus *Penicillium*. Fig 12 shows the outcome of lactophenol blue staining of some of the fungal isolates. Subsequent identification steps were only performed on those isolates that showed significant biodegradation effectiveness.

### Identification of bacterial and fungal isolates

The similarity of the sequences obtained against known deposited 18S rRNA and 16S rRNA sequences from closely related fungi and bacteria respectively was tested with BLASTN 2.2.1 upon sequence editing with Chromas Pro version 2.6.2 and accession numbers obtained from NCBI GenBank as shown (Tables 4 and 5).

**Table 4:** Percentage sequence similarity of fungal isolates with their closest taxonomic relatives in the NCBI GenBank.

No.	Sequence -ID	Isolate code	NCBI Accession number	Organism	% Similarity
1	2	E4,1	MG779504	*Aspergillus nidulans* strain voucher MF 109	100%
2	4	A1,1	MG779505	*Aspergillus insuetus* strain JAU1	100%
3	5	A4,2	MG779506	*Aspergillus flavus* strain AD-Jt-1	100%
4	6	C5,1	MG779507	*Aspergillus nidulans* strain Ya10	99%
5	8	A5,1	MG779508	*Aspergillus oryzae* strain RIB40	99%
6	10	D5,2	MG779509	*Aspergillus flavus* strain Ya1	100%
7	12	B5, 1	MG779510	*Aspergillus neoflavipes* strain AJR1	100%
8	14	E1,2	MG779511	*Aspergillus nidulans* strain FGSC A4	100%
9	15	D4,2	MG779512	*Aspergillus terreus* strain BTK-1	99%
10	16	B2,2	MG779513	*Aspergillus fumigatus* strain T3	99%

**Table 5:** Percentage sequence similarity of bacterial isolates with their closest taxonomic relatives in NCBI GenBank.

No.	Sequence -ID	Isolate code	NCBI Accession number	Organism	% Similarity
**1**	1	E1,2a	MG645252	*Bacillus cereus*	99%
**2**	2	B4,1 pn	MG645253	*Bacillus cereus*	99%
**3**	3	C2,2a	MG645254	*Brevibacillus parabrevis*	100%
**4**	4	B4, 2yn	MG645255	*Bacillus cereus*	99%
**5**	6	A5,a1	MG645256	*Bacillus cereus*	99%
**6**	7	D4, 1n	MG645257	*Bacillus toyonensis*	98%
**7**	9	E5,a1	MG645258	*Bacillus thuringiensis*	99%
**8**	10	B1,2	MG645259	*Bacillus thuringiensis*	99%
**9**	11	E4,1	MG645260	*Bacillus subtilis*	98%
**10**	12	D4,yn	MG645261	*Brevibacillus borstelensis*	99%
**11**	13	E4,1,2	MG645262	*Ochrobactrum pseudintermedium*	99%
**12**	14	C4,1a	MG645263	*Lysinibacillus macroides*	99%
**13**	15	A1, a	MG645264	*Bacillus cereus*	98%
**14**	17	A2,3	MG645265	*Bacillus pseudomycoides*	99%
**15**	19	C4,1a	MG645266	*Cellulosimicrobium funkei*	97%
**16**	20	B2,2	MG645267	*Brevibacillus borstelensis*	98%
**17**	24	B,4,2	MG645268	*Bacillus safensis*	99%
**18**	25	B,2,2a	MG645269	*Bacillus safensis*	99%
**19**	26	B1, 1a	MG645383	*Pseudomonas putida*	94%
**20**	29	C4,1a	MG645270	*Bacillus niacini*	98%

## Discussion

The bacterial inoculum of *Bacillus cereus* strain A5,a (MG64264) and *Brevibacillus borstelensis* strain B2,2 (MG645267) produced a mean weight loss of 35.72±4.01% and 20.28±2.30 respectively on the 30 micron polyethylene sheets (Table 1) which was significantly higher than the other bacterial samples. This is in agreement with the results recorded by [12] in which *Brevibacillus borstelensis* strain 707 after 30 days at 50°C reduced the gravimetric and molecular weights of polyethylene sheets by 11 and 30% respectively. The inoculum sample of *Pseudomonas putida* strain B1, 1a (MG645383) gave 2.80±0.38% mean weight loss on the 30 micron polythene. This was however a lower rate compared to the study done by [24] where *Pseudomonas sp* was subjected to LDPE biodegradation alongside three other genera of bacteria and it was the most effective bio-degrader. Fungal mean weight reductions was generally higher than bacterial with the highest mean weight reduction of 36.4±5.53%, 24±3.26% and 18±2.20% (Table 1) attributed to isolates *Aspergillus oryzae* strain A5,1(MG779508), *Aspergillus fumigatus* strain B2,2(MG779513) and *Aspergillus nidulans* E1,2 (MG779511) (Table 4) respectively. LDPE degradation by *Aspergillus* and *Bacillus* was recorded by [16]. However the mean weight reductions per sampling point were lower which is an indication that biodegradation of materials varies by point location depending on the microbial composition of the particular point. Use of weight reduction as a measure of the extent of polyethylene biodegradation has been widely accepted and used by many authors [25–27]. These outcomes are in agreement with [4] who reported the ability of microorganisms to degrade virgin polyethylene.

Analysis of the polyethylene spectral figures (Figs 7–11) indicate formation of new peaks at the region between 1700 and 1650. Also new peaks can be seen in the region between 1000 and 1100. The new peaks at 1700–1650 are indicative of formation of aldehydes and ketones which are intermediate products of biodegradation of polyethylene. The region of increased peak absorbance and new peaks in the 1000–1200 cm-1 region of the FTIR spectrum correlates with primary and secondary alcohols. The main bands of the studied LDPE sheets consist of a band situated about 2900 cm-1 assignable to CH2 as an asymmetric stretching, a band around 1461–1466 cm-1 revealing a bending deformation, and another band at 720–724 cm-1 which indicates a rocking deformation [28]. Intensity of the bands at 1650 cm−1 increased in the powder samples relative to the control. These results are however in contrast to the report by [29,30, 31] who concluded that microorganisms can only degrade chemically or physically pre-treated polyethylene. Presence of 4, 6-Octadiyn-3-one, 2-methyl which is a ketone in addition to 4,4-Dimethyl-2-pentene which is an alkene in the culture supernatant through GC-MS detection can be attributed to the process of biodegradation of the polymer where ketones are part of the intermediary products (Table 3). This was observed in just one of the samples that had been incubated with the consortium for degradation. This outcome is in agreement with a previous study by [15] who reported that large number of different aldehydes, ketones and carboxylic acids were identified in smoke generated on sheet extrusion of LDPE in an extrusion coating process. In the present study, the degraded products were determined by GC-MS analysis. The LDPE of 30 microns was better degraded than the 40 micron one due to its lower molecular weight while fungal rate of biodegradation was higher than bacterial biodegradation (Tables 1 and 2).

Among the isolates identified in this study were *Pseudomonas*, *Bacillus*, *Brevibacillus*, *Ochrobactrum*, *Lysinibacillus Cellulosimicrobium* and *Aspergillus* (Tables 4 and 5). [11] confirmed that Pseudomonas is a widely implicated bacterial genus in LDPE degradation and they also isolated a novel strain; *Pseudomonas citronellolis* EMBS027 as the most effective bio-degrader. According to [12], a thermophilic bacterium *Brevibaccillus borstelensis* strain 707 (isolated from soil) utilized LDPE as the sole carbon source.

## Conclusion and recommendations

The present work indicates that naturally growing soil microbes like bacteria and fungi show great efficacy in degrading polyethylene. Generally fungi have a higher degrading effectiveness compared to bacteria. However both fungi and bacteria showed capacity to degrade virgin polyethylene under laboratory conditions. Fungi of the genus *Aspergillus* and bacteria of the genus *Bacillus* had the highest capacity of degradation compared to the other genera in this study. Further efforts to improve this degrading capacity through assessment of optimum conditions for microbial activity so that this concept can be applied commercially and on a larger scale are necessary. Pre-treatment of polyethylene with substances that are environmentally friendly could also be adopted as a means to enhance polyethylene biodegradation.
